# Bacterial Calcium Carbonate Mineralization *in situ* Strategies for Conservation of Stone Artworks: From Cell Components to Microbial Community

**DOI:** 10.3389/fmicb.2020.01386

**Published:** 2020-06-30

**Authors:** Massimiliano Marvasi, Giorgio Mastromei, Brunella Perito

**Affiliations:** Department of Biology, University of Florence, Florence, Italy

**Keywords:** calcite biomineralization, biodeposition, bioremediation, stone conservation, stone microbiota, cultural heritage

## Abstract

Calcareous stones have been widely used in artworks and buildings by almost all human cultures. Now, more than ever, the increased environmental pollution and global warming are threatening the stone cultural heritage. Weathering due to physical, chemical and biological factors results in monumental calcareous stone deterioration. These agents induce a progressive dissolution of the mineral matrix, increase porosity, and lead to structural weakening. Bacterial Calcium Carbonate Mineralization is a widespread naturally occurring process which in the last decades was proposed as an environmentally friendly tool to protect monumental and ornamental calcareous stones. The advantage of this treatment is that it mimics the natural process responsible for stone formation, producing a mineral product similar to the stone substrate. This mini review highlights the milestones of the biomineralization approaches with focus on *in situ* stone artworks protection. The strategies explored to date are based on three main approaches: (i) the use of allochthonous and (ii) autochthonous alive cells that, due to the bacterial metabolism, foster biomineralization; (iii) the cell-free approach which uses fractionated cellular components inducing biomineralization. We discuss the challenging aspects of all these techniques, focusing on *in situ* applications and suggesting perspectives based on recent advances.

## Introduction

Bacterial Calcium Carbonate Mineralization (BCCM) is a widespread natural process of many bacterial taxonomic groups in different environments, ranging from microscopic crystals to large geological formations (Boquet et al., 1973; Ehrlich, 2002; Zavarzin, 2002; Dupraz et al., 2009; Perito and Mastromei, 2011).

According to Hammes and Verstraete (2002), BCCM is regulated by four key factors: calcium concentration, concentration of dissolved inorganic carbon (DIC), pH, and the availability of nucleation sites. Bacteria can foster an alkaline environment and increase DIC through different autotrophic and heterotrophic metabolic pathways (Castanier et al., 1999; Dhami et al., 2014; Zhu and Dittrich, 2016). If calcium ions and nucleation sites are available in the environment, BCCM then occurs.

Bacterial surfaces such as cell walls or esopolymeric substances (EPS), due to their metal binding properties, serve as nucleation sites and constitute particularly favorable templates for heterogeneous nucleation and crystal growth (Fortin et al., 1997; Douglas and Beveridge, 1998). The EPS act as matrix templates influencing CaCO_3_ crystal morphology, polymorphism, spatial position and growth (Braissant et al., 2003; Tourney and Ngwenya, 2009; Ercole et al., 2012; Oppenheimer-Shaanan et al., 2016). CaCO_3_ crystals usually grow on bacterial cell surfaces (Rivadeneyra et al., 1998; Castanier et al., 1999). The polymorph produced (mainly calcite, aragonite and vaterite) depends both on environmental conditions and bacterial strains (Ben Omar et al., 1997; Rivadeneyra et al., 1998; Brennan et al., 2004).

During the last decades, BCCM application was proposed as an environmentally friendly tool for conservation and reinforcement of monumental and ornamental calcareous stones (Orial et al., 1993). Weathering by physical, chemical and biological factors increases the porosity and dissolution of the mineral matrix thus progressively weakening the structure (Tiano et al., 1999). Organic products used to reduce monument deterioration present several drawbacks related to incompatibility with the stone, while inorganic consolidants show poor performance (De Muynck et al., 2010). The advantage of a BCCM-mediated treatment is that it mimics the natural process responsible for stone formation, producing a mineral product similar to the stone substrate. The aim is dual: to provide a coherent CaCO_3_ layer on the surface of deteriorated stone, protecting against the intake of water or chemicals, and to consolidate the inner, weakened structure. In literature a number of comprehensive reviews are available about biodeposition of CaCO_3_ on stone and building materials, highlighting mechanisms, limitations, challenges, and perspectives of this technology (De Muynck et al., 2010; Dhami et al., 2014; Anbu et al., 2016; Nazel, 2016; Zhu and Dittrich, 2016; Castro-Alonso et al., 2019). In this mini review, we fill a literature gap, by focusing on current BCCM technologies for *in situ* cultural stone conservation. We highlight the typology of interventions and recent improvements of *in situ* applications and provide viewpoints based on recent advances.

## BCCM-Based Approaches for Cultural Stone Conservation

### Living Cells, Single Selected Bacterial Strain

**FIGURE 1:**
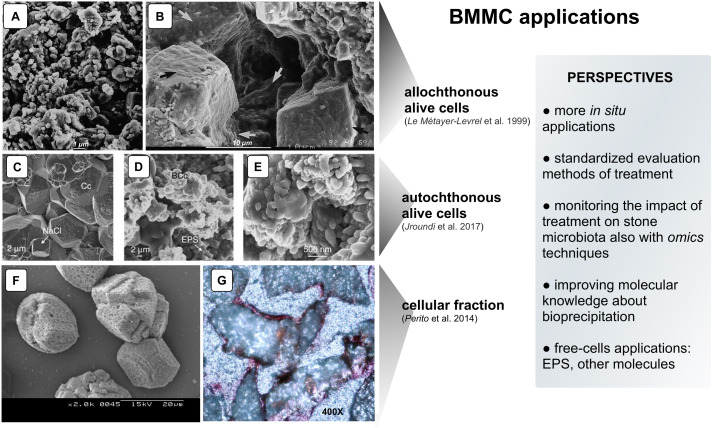
Examples of CaCO_3_ precipitation on limestone by different BCCM strategies. Panels **(A,B)** SEM micrographs of “biocalcin” formed by allochthonous alive cells on Saint Maxim (SM) limestone: **(A)** Untreated surface of SM limestone; **(B)** Pores filled with the superficial bacterial coating (arrows). Panels **(C–E)** SEM micrographs of calcite (determined by XRD) formed by autochthonous alive cells on calcarenite: **(C)** Untreated calcarenite. Chemically precipitated calcite crystals (Cc) in a control stone show dissolution pits and NaCl crystals; **(D)** In the treated stone, bacterial calcite (BCc) are organized in nanogranular structure surrounded by EPS; **(E)** Magnification of the nanogranular structure of calcite biocement. Panels **(F,G)** CaCO_3_ induced by cellular fraction: **(F)** SEM micrograph of calcite crystals (determined by XRD) induced by BCF in CaCl_2_ solution; **(G)** Representative thin section made from cores taken from stone slabs of the Angera Cathedral stained with Alizarine red (optical microscope, 400×). The metabolic pathway of allochthonous and autochthonous alive bacteria promoting BCCM (panels **A–E**) is the oxidative deamination of amino acids present in the nutrient medium. As a result, calcifying bacteria produce CO_2_ and NH_3_ creating an alkaline microenvironment and shifting the HCO_3_^–^ = CO_3_^2–^ + H^+^ equilibrium toward the right. In the presence of Ca^2+^, supplied in the nutrient medium, precipitation occurs via the reaction Ca^2+^ CO_3_^2^^−^ = CaCO_3_ preferentially on the bacterial cell surface in a microenvironment highly supersaturated with respect to CaCO_3_ (e.g., bacterial biofilm; Jroundi et al., 2017) (With permission from: Le Métayer-Levrel et al., 1999 for Panels **A,B**.; Jroundi et al., 2017 for Panels **C–E**; and Perito et al., 2014 for Panels **F,G**).

The application of BCCM for cultural heritage conservation was proposed by a pioneer French group that developed the so-called *Calcite Bioconcept* technology, covered by a now expired patent (Adolphe et al., 1990). This methodology was based on the application of cultures of selected bio-calcifying strains by spraying them on the stone surface and then feeding them by applications of a nutrient medium. The result was the formation of a new calcareous coating layer called *biocalcin.* This few μm thick layer was coherent to stone and made of encrusted bacterial bodies mixed with CaCO_3_ (Figures 1A,B). A preliminary screening of bacteria isolated from natural carbonate environments allowed the selection of a *Bacillus cereus* strain exhibiting the highest precipitation performance via the ammonification of amino acids (Table 1; Castanier et al., 2000). After testing it on limestone specimens, the technology was transferred to *in situ* applications (Le Métayer-Levrel et al., 1999). The first application was made in 1993, testing an area of 50 m^2^ of the tower of the Saint Médard Church in Thouars. Evaluation of the treatment was carried out 6 months and 1 year after the application (Table 1). The treatment had no influence on the color or other aesthetical features and the water absorption rate was up to five times less. Following this approach, a number of façades of French historic and private buildings were treated by the Calcite Bioconcept Company (Castanier et al., 2000; Anne et al., 2010; De Muynck et al., 2010). No scientific reports can be found about these treatments. At the same time, several groups have worked to improve this system by isolating and testing different microorganisms, exploring different metabolic pathways and application conditions mainly in laboratory settings, showing, in many cases, similar results (reviewed by Nazel, 2016).

**TABLE 1:**
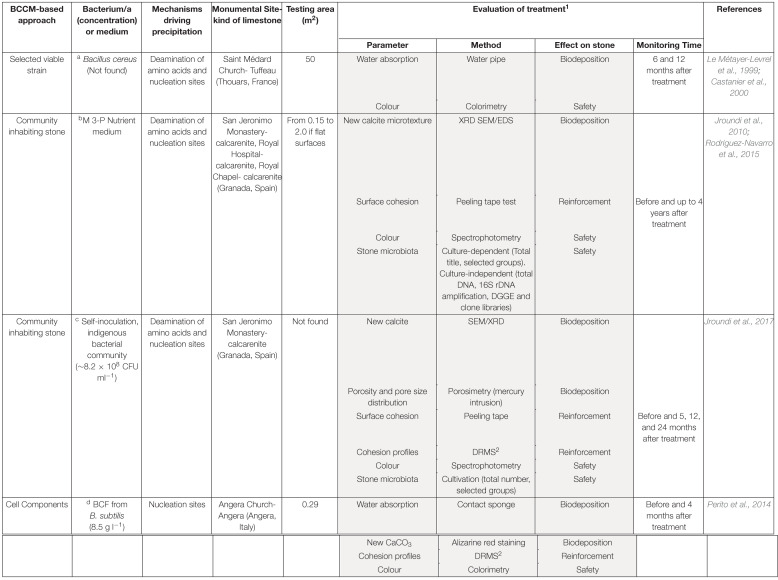
Features of *in situ* stone treatments of the three BCCM-based approaches. Details of the methodology of treatment, as found in the cited literature, are reported in the notes.

*^1^ The parameter assesses the effect of the treatment on: biodeposition, safety (condition of being protected from aesthetical, structural, or microbiological changes), and reinforcement. ^2^ Drilling Resistance Measurement System. ^a^The treatment is generically described in Le Métayer-Levrel et al. (1999). It consists of first spraying the entire surface to be protected with a suitable bacterial suspension culture. Afterward, the deposited culture is fed every 24–48 hours, usually for the next four days, with a suitable medium. ^b^ At the San Jeronimo Monastery and the Royal Hospital, the M-3P treatment was compared with the M. xanthus treatment. The treatments were applied by spray, twice a day, for 6 days (total volume applied 1–1.5 ml cm^–2^) on flat surfaces with similar wideness of calcarenite stone blocks. Treatment with M. xanthus involved two initial applications with inoculated M-3P (∼10^9^cells/ml), followed by successive applications with sterile M-3P solution. At the Royal Chapel, only the M-3P treatment was performed on elements of the crest with carved surfaces. In all cases, the treated area remained covered with an aluminum/plastic (bubble wrap) foil up to two/ three days after treatment, to avoid the direct effect of sunlight and to minimize evaporation (Rodriguez-Navarro et al., 2015). ^c^The self-inoculation treatment was compared with the M. xanthus and the sterile M-3P treatments. All three treatments were applied by spraying on adjacent stone blocks with similar exposure and decay levels. Approximately 0.125 ml cm^–2^were used for each application (twice a day for 6 days). In the M. xanthus and the self-inoculation treatments, the stone was treated on the first day with the bacterial culture and then with only the sterile M-3P nutritive solution. The treated areas were covered with an aluminum/ plastic foil up to 3 days after treatment (Jroundi et al., 2017). ^d^The field test was performed on the main façade of the Church of Angera. BCF in Super C solution) and REF (only Super C) were sprayed on the treated surface in selected areas of 0.29 m^2^and 0.28 m^2^, respectively, for a total application of 1 l m^–2^solution for each spray application. Spray application steps: the first day BCF solution at concentration of 8.5 g l^–1^in Super C and REF were sprayed, the second day 0.032 g l^–1^BCF in SuperC plus nanoparticles and REF plus nanoparticles were sprayed, while the third day only SuperC plus nanoparticles solution was sprayed on the selected areas (Perito et al., 2014).*

Over the last 20 years, a Spanish group of Granada made efforts to further develop this technology. They promoted the use of *Mixococcus xanthus*, a Gram-negative, non-pathogenic soil bacterium, to overcome drawbacks of previous treatments: the thin layer of the new formed bio-cement, the possible formation of endospores, and uncontrolled biofilm by *Bacillus* clogging stone pores. In an *in vitro* model, sterilized calcarenite slabs were immersed in a liquid medium containing *M. xanthus* and nutrients activating the ammonification of amino acids (Rodriguez-Navarro et al., 2003). Newly formed coherent carbonate cement of calcite grains was deposited into the pores without plugging them to a depth ≥500 μm. No myxospore formation was found in the tested culture media.

### Living Cells, Microbial Community of Stone

A further step in the development of this technology proposed by Jimenez-Lopez et al. (2007) was bio-precipitation fostered by the microbial community inhabiting the stone. The advantage was that it supported the autochthonous CaCO_3_ producing-bacteria without introducing exogenous microorganisms. Initially, quarry porous limestone slabs were immersed in a M-3P nutritive buffered solution with/without *M. xanthus* (Jimenez-Lopez et al., 2008). Treated stones showed newly precipitated CaCO_3_ overgrowth without pore plugging and, accordingly, weight increase, regardless of the presence or absence of *M. xanthus*. In comparison to sterilized slabs used as controls, the treated slabs maintained their original pore size distribution and were more resistant to mechanical stress. The M-3P medium, stimulating heterotrophic carbonatogenic bacteria via the ammonification of amino acids (Table 1), was patented (González-Muñoz et al., 2008).

The M-3P treatment was then tested *in situ*, with and without *M. xanthus*, on selected areas of decayed calcarenite stone of three historic buildings in Granada: San Jeronimo Monastery, Hospital Real and Royal Chapel (Jroundi et al., 2010; Rodriguez-Navarro et al., 2015). The evaluation included both the technical efficacy and, for the first time, the monitoring of the bacterial community of the decayed stone by culture-dependent and independent techniques (Table 1). Medium/long-term efficacy and detrimental side-effects were monitored up to 4 years after treatments (Rodriguez-Navarro et al., 2015). In all the three cases, the newly formed CaCO_3_ (mostly calcite) created a cement that consolidated the deteriorated calcarenite with a significant surface strengthening neither plugging pores nor causing aesthetical changes. The efficacy of the treatment *in situ* was independent of the presence of *M. xanthus*. The carbonatogenic bacterial population initially increased after treatment applications, but over time reached values close to those observed before treatment.

In those cases where the stone microbiota was altered and/or suppressed (e.g., application of biocides), the same authors proposed a bioconsolidation treatment with carbonatogenic bacteria selected from calcareous stones as inoculants (Jroundi et al., 2012). Bacteria were isolated from altered calcarenite stone slabs by the application of M-3P medium, then precipitating bacteria belonging to Actinobacteria, Gamma-proteobacteria and Firmicutes were selected and single strains were tested for bio-consolidation capability *in vitro*, with and without *M. xanthus.* They found that *Acinetobacter* spp. strains were the most appropriate candidate bacteria.

To test the self-inoculation biotreatment *in situ*, an indigenous community was recovered by cultivation from salt damaged carbonate stone in a historic building (San Jeronimo Monastery), activated via M-3P, and applied back onto the same stone (Jroundi et al., 2017). Firmicutes was the dominant phylum in the inoculum (∼79%). Test evaluation methods are reported in Table 1. The effective consolidation was due to the formation of an abundant and exceptionally strong hybrid cement consisting of nanostructured CaCO_3_ and bacterial EPS covering the substrate (Figures 1C–E). After 5 months, the viable titer of culturable microbiota increased and then after 24 months dropped back to about pre-treatment values.

### Cell Components

An Italian team of Florence investigated and assessed CaCO_3_ mineralization on stone induced by a bacteria-mediated system in absence of viable cells (Perito et al., 2014). This investigation used the *Bacillus subtilis* strain 168 to identify bacterial structures or molecules inducing precipitation. The precipitation capability of bacterial dead cells was tested in a CaCl_2_ solution as calcium source and with the sublimation of ammonium carbonate for alkalization. Dead cells were able to promote calcite formation, then cell fractions were tested and a bacterial cell fraction (BCF) containing the cell wall induced CaCO_3_ formation (Figure 1F). Interestingly, the system was specific in generating crystal polymorphisms, since only calcite was found by X-ray diffraction.

Apparently, dead cells as well as BCF acted as crystallization nuclei in liquid medium. This hypothesis is supported by the capacity of cell walls to uptake cations such as Ca^2+^, as previously demonstrated for isolated *B. subtilis* walls (Beveridge and Murray, 1980), and fostering heterogeneous nucleation (Fortin et al., 1997). According to Dupraz et al. (2009), this process can be referred to biologically influenced mineralization.

BCF was stored as easy-to use lyophilized preparations, maintained a long-lasting activity and showed heat resistance. BCF treatment was tested on slab stones and then *in situ* on selected areas of the main façade of the Angera Cathedral, a 6th century monumental site in Italy (Perito et al., 2014). Lyophilized BCF was dissolved in a CaCl_2_ solution, then sprayed on stone surface with a supersaturated calcium bicarbonate Ca(HCO_3_)_2_ solution (Super C solution) for supplying calcium ions and CO_2_. The solution was supplemented with calcite nanoparticles to maintain supersaturation in the pore and increase calcium ions. Field evaluation tests after treatment showed that BCF treated areas had negligible color changes (Table 1). New crystals formed inside stone pores (Figure 1G) and, accordingly, there was a significant decrease in water absorption (up to 6.8%). The cohesion profiles were significantly increased in the first 3 mm (if compared with the control area treated with Super C alone). These results show that this application has potential, even if the authors concluded that further testing was needed to fully assess the treatment conditions for *in situ* applications.

### Perspectives: From Cell Components to the Microbial Community

BCCM biotechnology could be an ecological alternative to chemical treatments due to the low environmental impact and the production of a layer of CaCO_3_ compatible with and coherent to the stone. A common point of improvement for the BCCM technologies is the consolidation performance, not yet comparable to that of synthetic polymers. The appropriate selection of stone types before application is important because pore structure affects penetration depth and treatment performance (De Muynck et al., 2011). Nanomechanical properties of CaCO_3_ polymorphs can also be improved by a better understanding of the bio-geo-chemical processes governing the formation of biominerals with high mechanical performance in natural environments (Dhami et al., 2018).

While the literature shows the potential in the laboratory of different bacterial applications to promote CaCO_3_ mineralization, very few attempts have been made to test the technology *in situ* (Table 1). Based on these few studies, some companies have developed biomineralization products for cultural heritage by using cultures of selected strains (Amonit, France^1^) or media stimulating stone microbiota (KBYO Biological, Spain^2^). Nevertheless, the translation of other promising results obtained *in vitro* into practical bioremediation applications on heritage stone *in situ* remains the challenge for the immediate future (Webster and May, 2006); as examples, the well documented biocalcite production by bacterial urease or carbonic anhydrase (Castro-Alonso et al., 2019). Scaling up will be needed in order to develop this technology (Figure 1, box perspectives). *In situ* applications always have additional problems when compared with the *in vitro* conditions, especially concerning heterogeneity and conservation state of the stone, delivery systems, outdoor or indoor environmental conditions, type of feasible evaluation tests and the value of the artwork. For this reason, preliminary *in situ* small-scale testing should adopt treatment conditions mimicking those to the follow in larger-scale applications.

Another general comment concerns the heterogeneity of the treatment evaluation tests (Table 1). Although the choice of the monitoring methods sometimes depends on the experimental set-up, evaluating methods must be rapidly standardized for comparing results and for metadata analyses. Standard methods should consider the effectiveness of the treatment in terms of both consolidation and safety of stone (impact on structural and aesthetical features as well as on resident microbiota).

Living bacteria require the application of nutrient media on the stone. The possibility of undesirable side-effects on stone is controversial and it needs to be carefully evaluated (González-Muñoz, 2008; Nazel, 2016). The metabolic pathway activated *in situ* is the oxidative deamination of amino acids (Table 1), which increases the alkalinity by production of ammonia (Castanier et al., 1999; Lee and Park, 2019). The convenience of obtaining byproducts as ammonia and using spore-forming bacteria as *Bacillus* on stone has been recently discussed (Dhami et al., 2014; Zhu and Dittrich, 2016). More generally, promotion of undesired microbial growth can produce mineral changes or appearance of stained patches on stone, as found by Tiano et al. (1999). Such drawbacks may be encountered both in case of activating allochthonous or autochthonous strains. While aesthetical changes can be easily evaluated, growth of unwanted microorganisms and/or changes in the autochthonous community structure affecting the original ecological niche is harder to analyze. Microbes can strongly contribute to stone deterioration (Pinna, 2017) and the application of new biotechnologies by conservators requires knowledge about the risk factors, in particular on the long-term effects (Webster and May, 2006; De Muynck et al., 2010). In this respect, the work about the long-term monitoring of stone microbiota carried by Ettenauer et al. (2011) and Jroundi et al. (2017) is remarkable. However, knowledge about microbial communities inhabiting heritage stone mainly comes from cultivation studies (Scheerer et al., 2009). Microbial communities of stone were only recently investigated using Next Generation Sequencing and omics techniques (Perito and Cavalieri, 2018; Marvasi et al., 2019). The latest studies suggest that natural community structure detected by metagenomics is quite different from that of enriched communities cultivated from calcareous stone in precipitating media where Firmicutes are dominant (Dhami et al., 2018; Li et al., 2018).

Meta-omics techniques as a whole (metagenomics, metatranscriptomics and metabolomics) will promote a further step to improving BCCM technology, because they provide a wider view of the microbial community structure, fluctuations and metabolic potential (Marvasi et al., 2019). In regard to the cultivation bias (Hardoim et al., 2014), omics technologies will provide a better understanding of the stone microbial community structure to allow treatment monitoring as well as the identification of the community components with biomineralization potential. Chimienti et al. (2016) used metagenomics to identify the presence of microorganisms known as carbonatogenic (i.e., *Arthrobacter*) within the overall microbial community from stone slabs of a medieval church. Zanardini et al. (2019) reconstructed the carbon, nitrogen and sulfur cycles and their biodeterioration potential within the prokaryotic community of decayed sandstone of a medieval castle by 16S rRNA and functional gene analyses. Using a similar approach, the carbonatogenic potential of metabolic pathways linked to these biogeochemical cycles could be inferred. On the other hand, cultivation is more valuable than ever in the omics era (Gutleben et al., 2018) because it is needed to confirm the predicted carbonatogenic ability of stone populations as well as for other applications. But then again, meta-omics techniques can also provide useful information to improve cultivation strategies for the isolation of potential calcinogenic bacterial populations from calcareous environments.

The cell-free approach offers several advantages: the cellular components act as mineral nucleation and growth sites in the absence of nutrients, components smaller than cells penetrate more in depth into pores and microcracks, interventions on the chemical environment governing precipitation are easier (Hammes and Verstraete, 2002). Alkaline buffering or different supersaturated calcium solutions should be further developed and compared to that used by Perito et al. (2014). However, the preparation of the BCF product is more complex compared to alive cellular strategies but could have as target calcareous objects where minimum change in their chemistry is required (Perito et al., 2014). A cell-free approach has not been explored further.

Very little is still known about the molecular basis of the calcium biomineralization process (Perito and Mastromei, 2011). *B. subtilis* laboratory strain 168 was used to identify cellular fractions as well as genes and molecules with key roles in inducing precipitation (Barabesi et al., 2007), as found for mollusks (Falini et al., 1996). Characterization of *B. subtilis* mutants impaired in CaCO_3_ precipitation suggested a link between biomineralization, redox reactions of fatty acid metabolism, changes in phospholipids membrane composition and surface properties (Barabesi et al., 2007; Marvasi et al., 2010, 2016; Frandi et al., 2011; Perito et al., 2018a). In *Lysinibacillus*, CaCO_3_ precipitation can modify membrane rigidity by upregulating the branched chain fatty acid synthesis (Lee and Park, 2019). We speculate that intervention on these metabolic switches could help in the search for bacterial molecules fostering precipitation and, at the same time, improving precipitation performance by bacteria.

On the other hand, it is well known that bacterial macromolecules, like the EPS, act as matrices which promote mineralization and are trapped in the growing calcite (Decho, 2010; Marvasi et al., 2012; Perito et al., 2018b). According to Jroundi et al. (2017), the hybrid cement due to the incorporation of organisms and EPS within the nanostructured CaCO_3_ in the self-inoculation biotreatment was responsible for the high consolidation effectiveness. Further studies are needed in order to identify and test different EPS or to design bacteria-based biomimetic matrices promoting calcite growth on stone. This would represent a further advancement of the cell-free technology since it would reduce the complexity of organic matter to apply, increasing its penetration inside stone.

Concluding, in our opinion all the different approaches explored in this mini review are worth further development for *in situ* applications, even if two of them are already available on the market. Fascinating challenges for the future include advances in exploitation of bacterial pathways, cell components and single (macro)molecules.

## Author Contributions

BP provided the general concept. BP and MM wrote the manuscript. MM, GM, and BP revised and approved the manuscript. All authors contributed to the article and approved the submitted version.

## Conflict of Interest

The authors declare that the research was conducted in the absence of any commercial or financial relationships that could be construed as a potential conflict of interest. The reviewer CO declared a past collaboration with one of the authors BP to the handling Editor.

## Footnotes

1 http://www.amonit.fr/fr/calcite__1

2 http://kbyobiological.com/en/

## References

[B1] Adolphe J. P. Loubière J. F. Paradas J. Soleilhavoup F. (1990). *Procédé De Traitement Biologique D’une Surface Artificielle.* European patent 90400G97.0.

[B2] Anbu P. Kang C. H. Shin Y. J. So J. S. (2016). Formations of calcium carbonate minerals by bacteria and its multiple applications. *Springerplus* 5:250 10.1186/s40064-016-1869-2 PMC4771655 27026942

[B3] Anne S. Rozenbaum O. Andreazza P. Rouet J.-L. (2010). Evidence of a bacterial carbonate coating on plaster samples subjected to the calcite bioconcept biomineralization technique. *Constr. Build. Mater.* 24 1036–1042. 10.1016/j.conbuildmat.2009.11.014

[B4] Barabesi C. Galizzi A. Mastromei G. Rossi M. Tamburini E. Perito B. (2007). *Bacillus subtilis* gene cluster involved in calcium carbonate biomineralization. *J. Bacteriol.* 189 228–235. 10.1128/JB.01450-06 17085570 PMC1797216

[B5] Ben Omar N. Arias J. M. Gonzalez-Munoz M. T. (1997). Extracellular bacterial mineralization within the context of geomicrobiology. *Microbiologia* 13 61–72. 9253756

[B6] Beveridge T. J. Murray R. G. E. (1980). Sites of metal deposition in the cell wall of *Bacillus subtilis*. *J. Bacteriol.* 141 876–887. 10.1128/jb.141.2.876-887.1980 6767692 PMC293699

[B7] Boquet E. Boronat A. Ramos-Cormenzana A. (1973). Production of calcite (calcium carbonate) crystals by soil bacteria is a general phenomenon. *Nature* 246 527–529. 10.1038/246527a0

[B8] Braissant O. Cailleau G. Dupraz C. Verrecchia E. P. (2003). Bacterially induced mineralization of calcium carbonate in terrestrial environments: the role of exopolysaccharides and amino acids. *J. Sediment Res.* 73 485–490. 10.1306/111302730485

[B9] Brennan S. T. Lowenstein T. K. Horita J. (2004). Seawater chemistry and the advent of biocalcification. *Geology* 32 473–476. 10.1130/G20251.1

[B10] Castanier S. Le Métayer-Levrel G. Orial G. Loubière J. F. Perthuisot J. P. (2000). “Bacterial carbonatogenesis and applications to preservation and restoration of historic property,” in *Of Microbes And Art: The Role Of Microbial Communities In The Degradation And Protection Of Cultural Heritage*, eds Ciferri O. Tiano P. Mastromei G. (New York, NY: Plenum Publishers), 203–218. 10.1007/978-1-4615-4239-1_14

[B11] Castanier S. Le Métayer-Levrel G. Perthuisot J. P. (1999). Ca-carbonates precipitation and limestone genesis - the microbiogeologist point of view. *Sediment Geol.* 126 9–23. 10.1016/S0037-0738(99)00028-7

[B12] Castro-Alonso M. J. Montañez-Hernandez L. E. Sanchez-Muñoz M. A. Macias Franco M. R. Narayanasamy R. Balagurusamy N. (2019). Microbially induced calcium carbonate precipitation (MICP) and its potential in bioconcrete: microbiological and molecular concepts. *Front. Mater.* 6:126 10.3389/fmats.2019.00126

[B13] Chimienti G. Piredda R. Pepe G. van der Werf I. D. Sabbatini L. Crecchio C. (2016). Profile of microbial communities on carbonate stones of the medieval church of San leonardo di siponto (Italy) by Illumina-based deep sequencing. *Appl. Microbiol. Biotechnol.* 100 8537–8548. 10.1007/s00253-016-7656 27283019

[B14] De Muynck W. De Belie N. Verstraete W. (2010). Microbial carbonate precipitation in construction materials: a review. *Ecol. Eng.* 36 118–136. 10.1016/j.ecoleng.2009.02.006

[B15] De Muynck W. Leuridan S. Van Loo D. Verbeken K. Cnudde V. De Belie N. (2011). Influence of pore structure on the effectiveness of a biogenic carbonate surface treatment for limestone conservation. *Appl. Environ. Microbiol.* 77 6808–6820. 10.3389/fmicb.2014.00304 21821746 PMC3187100

[B16] Decho A. W. (2010). Overview of biopolymer-induced mineralization: what goes on in biofilms? *Ecol. Eng.* 36 137–144. 10.1016/S0168-6496(98)00027-0

[B17] Dhami N. K. Mukherjee A. Watkin E. L. J. (2018). Microbial diversity and mineralogical-mechanical properties of calcitic cave speleothems in natural and in vitro biomineralization conditions. *Front. Microbiol.* 9:40 10.3389/fmicb.2018.00040 PMC5810276 29472898

[B18] Dhami N. K. Reddy M. S. Mukherjee A. (2014). Application of calcifying bacteria for remediation of stones and cultural heritages. *Front. Microbiol.* 5:304 10.3389/fmicb.2014.00304 PMC4071612 25018751

[B19] Douglas S. Beveridge T. J. (1998). Mineral formation by bacteria in natural microbial communities. *FEMS Microb. Ecol.* 26 79–88. 10.1111/j.1574-6941.1998.tb00494.x

[B20] Dupraz C. Reid R. P. Braissant O. Decho A. W. Norman R. S. Visscher P. T. (2009). Processes of carbonate precipitation in modern microbial mats. *Earth Sci. Rev.* 96 141–162. 10.1016/j.earscirev.2008.10.005

[B21] Ehrlich H. L. (2002). *Geomicrobiology*, 4th Edn, New York, NY: Marcel Dekker.

[B22] Ercole C. Bozzelli P. Altieri F. Cacchio P. Del Gallo M. (2012). Calcium carbonate mineralization: involvement of extracellular polymeric materials isolated from calcifying bacteria. *Micros. Microanal.* 18 829–839. 10.1017/S1431927612000426 22697480

[B23] Ettenauer J. Piñar G. Sterflinger K. Gonzalez-Muñoz M. T. Jroundi F. (2011). Molecular monitoring of the microbial dynamics occurring on historical limestone buildings during and after the in situ application of different bio-consolidation treatments. *Sci. Total Environ.* 409 5337–5352. 10.1016/j.scitotenv.2011.08.063 21944202 PMC3209562

[B24] Falini G. Albeck S. Weiner S. Addadi L. (1996). Control of aragonite or calcite polymorphism by mollusk shell macromolecules. *Science* 271 67–69. 10.1126/science.271.5245.67

[B25] Fortin D. Ferris F. G. Beveridge T. J. (1997). Surface-mediated mineral development by bacteria. *Rev. Min. Geochem.* 35 161–180. 10.1515/9781501509247-007

[B26] Frandi A. Zucca P. Marvasi M. Mastromei G. Sanjust E. Perito B. (2011). *Bacillus subtilis* fadB (ysiB) gene encodes an enoyl-CoA hydratase. *Ann. Microbiol.* 61 371–374. 10.1007/s13213-010-0121-5

[B27] González-Muñoz M. T. (2008). Bacterial biomineralization applied to the protection-consolidation of ornamental stone: current development and perspectives. *Coalition* 15 12–18.

[B28] González-Muñoz M. T. Rodriguez-Navarro C. Jimenez-Lopez C. Rodriguez-Gallego M. (2008). *Method and Product For Protecting And Reinforcing Construction And Ornamental Materials, Publication Number.* Spanish patent P200602030 (WO2008009771A1).

[B29] Gutleben J. De Mares M. C. van Elsas J. D. Smidt H. Overmann J. Sipkema D. (2018). The multi-omics promise in context: from sequence to microbial isolate. *Crit. Rev. Microbiol.* 44 212–229. 10.1080/1040841X.2017.1332003 28562180

[B30] Hammes F. Verstraete W. (2002). Key roles of pH and calcium metabolism in microbial carbonate precipitation. *Rev. Environ. Sci. Biotechnol.* 1 3–7. 10.1023/A:1015135629155

[B31] Hardoim C. C. P. Cardinale M. Cucio A. C. B. Esteves A. I. S. Berg G. Xavier J. R. (2014). Effects of sample handling and cultivation bias on the specificity of bacterial communities in keratose marine sponges. *Front. Microbiol.* 5:611 10.3389/fmicb.2014.00611 PMC4235377 25477868

[B32] Jimenez-Lopez C. Jroundi F. Pascolini C. Rodriguez-Navarro C. Piñar G. Rodriguez-Gallego M. (2008). Consolidation of quarry calcarenite by calcium carbonate precipitation induced by bacteria activated among the microbiota inhabiting the stone. *Int. Biodeter. Biodegrad.* 62 352–363. 10.1016/j.ibiod.2008.03.002

[B33] Jimenez-Lopez C. Rodriguez-Navarro C. Pióar G. Carrillo-Rosa F. J. Rodriguez-Gallego M. Gonzalez-Muñoz M. T. (2007). Consolidation of degraded ornamental porous limestone stone by calcium carbonate precipitation induced by the microbiota inhabiting the stone. *Chemosphere* 68 1929–1936. 10.1016/j.chemosphere.2007.02.044 17418886

[B34] Jroundi F. Fernandez-Vivas A. Rodriguez-Navarro C. Bedmar E. J. Gonzalez-Muñoz M. T. (2010). Bioconservation of deteriorated monumental calcarenite stone and identification of bacteria with carbonatogenic activity. *Microb. Ecol.* 60 39–54. 10.1007/s00248-010-9665-y 20386895

[B35] Jroundi F. Gómez-Suaga P. Jimenez-Lopez C. González-Muñoz M. T. Fernandez-Vivas M. A. (2012). Stone-isolated carbonatogenic bacteria as inoculants in bioconsolidation treatments for historical limestone. *Sci. Total Environ.* 425 89–98. 10.1016/j.scitotenv.2012.02.059 22464961

[B36] Jroundi F. Schiro M. Ruiz-Agudo E. Elert K. Martín-Sánchez I. González-Muñoz M. T. (2017). Protection and consolidation of stone heritage by self-inoculation with indigenous carbonatogenic bacterial communities. *Nat. Commun.* 8:279 10.1038/s41467-017-00372-3 PMC5561188 28819098

[B37] Le Métayer-Levrel G. Castanier S. Orial G. Loubière J. F. Perthuisot J. P. (1999). Application of bacterial carbonatogenesis to the protection and regeneration of limestones in buildings and historical patrimony. *Sediment Geol.* 126 25–34. 10.1016/S0037-0738(99)00029-9

[B38] Lee Y. S. Park W. (2019). Enhanced calcium carbonate-biofilm complex formation by alkali-generating *Lysinibacillus boronitolerans* YS11 and alkaliphilic *Bacillus* sp. AK13. *AMB Expr.* 9:49 10.1186/s13568-019-0773-x PMC6459448 30976947

[B39] Li Q. Zhang B. Yang X. Ge Q. (2018). Deterioration-associated microbiome of stone monuments: structure, variation, and assembly. *Appl. Environ. Microbiol.* 84:e2680-17 10.1128/AEM.02680-17 PMC5861828 29374040

[B40] Marvasi M. Casillas-Santiago L. M. Henríquez T. Casillas-Martinez L. (2016). Involvement of etfA gene during CaCO3 precipitation in *Bacillus subtilis* biofilm. *Geomicrobiol. J.* 34 722–728. 10.1080/01490451.2016.1248254

[B41] Marvasi M. Cavalieri D. Mastromei G. Casaccia A. Perito B. (2019). Omics technologies for an in-depth investigation of biodeterioration of cultural heritage. *Int. Biodeter. Biodeg.* 144:104736 10.1016/j.ibiod.2019.104736

[B42] Marvasi M. Gallagher K. L. Martinez Casillas L. Molina Pagan W. C. Rodrıguez Santiago R. E. Castilloveitía Vega G. (2012). Importance of B4 medium in determining organomineralization potential of bacterial environmental isolates. *Geomicrobiol. J.* 29 916–924. 10.1080/01490451.2011.636145

[B43] Marvasi M. Visscher P. T. Perito B. Mastromei G. Casillas-Martinez L. (2010). Physiological requirements for carbonate precipitation during biofilm development of *Bacillus subtilis* etfA mutant. *FEMS Microbiol Ecol.* 71 341–350. 10.1111/j.1574-6941.2009.00805.x 20059546

[B44] Nazel T. (2016). Bioconsolidation of stone monuments. An overview. *Rest. Build. Monum.* 22 37–45. 10.1515/rbm-2016-0001

[B45] Oppenheimer-Shaanan Y. Sibony-Nevo O. Bloom-Ackermann Z. Suissa R. Steinberg N. Kartvelishvily E. (2016). Spatio-temporal assembly of functional mineral scaffolds within microbial biofilms. *NPJ Biofilms Microb.* 2016:15031 10.1038/npjbiofilms.2015.31 PMC5515261 28721240

[B46] Orial G. Castanier S. Le Métayer-Levrel G. Loubiere J. F. (1993). “The biomineralization: a new process to protect calcareous stone applied to historic monuments,” in *Proceedings of the 2nd International Conference Biodeterioration of Cultural Property*, eds Toishi H. K. Arai T. Kenjo K. (Tokyo: International Communications Specialists), 98–116.

[B47] Perito B. Casillas L. Marvasi M. (2018a). Factors affecting formation of large calcite crystals (=1mm) in *Bacillus subtilis* 168 biofilm. *Geomicrobiol. J.* 35 385–391. 10.1080/01490451.2017.1377788

[B48] Perito B. Romanelli M. Buccianti A. Passaponti M. Montegrossi G. Di Benedetto F. (2018b). An XRPD and EPR spectroscopy study of microcrystalline calcite bioprecipitated by *Bacillus subtilis*. *Phys. Chem. Miner.* 45 935–944. 10.1007/s00269-018-0974-x

[B49] Perito B. Cavalieri D. (2018). Innovative metagenomic approaches for detection of microbial communities involved in biodeteriorattion of cultural heritage. *IOP Conf. Ser. Mater. Sci. Eng.* 364:012074 10.1088/1757-899X/364/1/012074

[B50] Perito B. Marvasi M. Barabesi C. Mastromei G. Bracci S. Vendrell M. (2014). A *Bacillus subtilis* cell fraction (BCF) inducing calcium carbonate precipitation: biotechnological perspectives for monumental stone reinforcement. *J. Cult. Herit.* 15 345–351. 10.1016/j.culher.2013.10.001

[B51] Perito B. Mastromei G. (2011). Molecular basis of bacterial calcium carbonate precipitation. *Prog. Mol. Subcell Biol.* 52 113–139. 10.1007/978-3-642-21230-7_5 21877265

[B52] Pinna D. (2017). *Coping with Biological Growth On Stone Heritage Objects: Methods, Products, Applications, And Perspectives.* Oakville, ON: Apple Academic Press Inc.

[B53] Rivadeneyra M. A. Delgado G. Ramos-Cormenzana A. Delgado R. (1998). Biomineralization of carbonates by *Halomonas eurihalina* in solid and liquid media with different salinities: crystal formation sequence. *Res. Microbiol.* 149 277–287. 10.1016/S0923-2508(98)80303-3 9766229

[B54] Rodriguez-Navarro C. Jroundi F. Gonzalez-Muñoz M. T. (2015). Stone consolidation by bacterial carbonatogenesis: evaluation of in situ application. *Restor. Build. Monum.* 21 9–20. 10.1515/rbm-2015-0002

[B55] Rodriguez-Navarro C. Rodriguez-Gallego M. Ben Chekroun K. Gonzalez-Muñoz M. T. (2003). Conservation of ornamental stone by *Myxococcus xanthus*-induced carbonate biomineralization. *Appl. Environ. Microbiol.* 69 2182–2193. 10.1128/AEM.69.4.2182-2193.2003 12676699 PMC154787

[B56] Scheerer S. Ortega-Morales O. Gaylarde C. (2009). Microbial deterioration of stone monuments-an updated overview. *Adv. Appl. Microbiol.* 66 97–139. 10.1016/S0065-2164(08)00805-8 19203650

[B57] Tiano P. Biagiotti L. Mastromei G. (1999). Bacterial bio-mediated calcite precipitation for monumental stones conservation: methods of evaluation. *J. Microbiol. Methods* 36 139–145. 10.1016/S0167-7012(99)00019-6 10353808

[B58] Tourney J. Ngwenya B. T. (2009). Bacteria extracellular polymeric substance (EPS) mediate CaCO3 morphology and polymorphism. *Chem. Geol.* 262 138–146. 10.1016/j.chemgeo.2009.01.006

[B59] Webster A. May E. (2006). Bioremediation of weathered-building stone surfaces. *Trends Biotechnol.* 24 255–260. 10.1016/j.tibtech.2006.04.005 16647149

[B60] Zanardini E. May E. Purdy K. J. Murrel J. C. (2019). Nutrient cycling potential within microbial communities on culturally important stoneworks. *Environ. Microbiol. Rep.* 11 147–154. 10.1111/1758-2229.12707 30346661 PMC7379959

[B61] Zavarzin G. A. (2002). Microbial geochemical calcium cycle. *Microbiology* 71 1–17. 11910807

[B62] Zhu T. Dittrich M. (2016). Carbonate precipitation through microbial activities in natural environment, and their potential in biotechnology: a review. *Front. Bioeng. Biotechnol.* 4:4 10.3389/fbioe.2016.00004 PMC4718973 26835451

